# Object Recognition in Mental Representations: Directions for Exploring Diagnostic Features through Visual Mental Imagery

**DOI:** 10.3389/fpsyg.2017.00833

**Published:** 2017-05-23

**Authors:** Stephanie M. Roldan

**Affiliations:** Virginia Tech Visual Neuroscience Laboratory, Psychology Department, Virginia Polytechnic Institute and State University, BlacksburgVA, United States

**Keywords:** object recognition, diagnostic features, mental imagery, visual perception, neuroimaging, eye tracking

## Abstract

One of the fundamental goals of object recognition research is to understand how a cognitive representation produced from the output of filtered and transformed sensory information facilitates efficient viewer behavior. Given that mental imagery strongly resembles perceptual processes in both cortical regions and subjective visual qualities, it is reasonable to question whether mental imagery facilitates cognition in a manner similar to that of perceptual viewing: via the detection and recognition of distinguishing features. Categorizing the feature content of mental imagery holds potential as a reverse pathway by which to identify the components of a visual stimulus which are most critical for the creation and retrieval of a visual representation. This review will examine the likelihood that the information represented in visual mental imagery reflects distinctive object features thought to facilitate efficient object categorization and recognition during perceptual viewing. If it is the case that these representational features resemble their sensory counterparts in both spatial and semantic qualities, they may well be accessible through mental imagery as evaluated through current investigative techniques. In this review, methods applied to mental imagery research and their findings are reviewed and evaluated for their efficiency in accessing internal representations, and implications for identifying diagnostic features are discussed. An argument is made for the benefits of combining mental imagery assessment methods with diagnostic feature research to advance the understanding of visual perceptive processes, with suggestions for avenues of future investigation.

## Introduction

Traditional object recognition research frequently focuses on bottom–up processing of visual stimuli, proceeding from the detection of stimulus properties by the retinal cells to electrical transduction and consummate neural response. This vein of research has been successful in identifying physiological and neural pathways involved in the detection and processing of visual object properties leading to cognitive perception. Visual mental imagery therefore represents a flow of information opposite to that of visual perceptual phenomena; one which requires a departure from traditional bottom–up perspectives in order to be fully understood.

With the advent of enhanced technologies and improved testing methods, visual mental imagery research has progressed from mere speculation on a largely inaccessible, theoretical phenomenon to an effective and valid field of research with a rich empirical background. Increasing numbers of studies have demonstrated the functional role of visual imagery in a variety of tasks, such as memory (Slotnick et al., 2005; Albers et al., 2013), creative design (Dahl et al., 1999; D’Ercole et al., 2010), and emotional disorders (Holmes and Mathews, 2010). In a recent review, Pearson et al. (2015) expounded the very practical implications of mental imagery in mental illness research and treatment, resulting in a call to advance the pursuit of mental imagery as a prime target of psychopathological interventions. Discussions such as these not only illustrate the evolving attitude toward the significance of mental imagery, but also emphasize the potential benefits of further exploration into this complex process. However, despite the significant growth undergone by this area of research, the current understanding of mental imagery often remains limited to general operations and characteristics; in both colloquial and scientific terms, visual mental imagery commonly serves as a broad and somewhat abstract definition of any visual substance that exists within the “mind’s eye.” But what exactly does one “see” during imaginary experiences? *Why* are certain images, or subcomponents of images, visualized more clearly than others, and what does this suggest about the process of perceptual viewing? Such questions remain to be answered in any conclusive or specific manner, and it is the goal of this review to evaluate possible avenues for working toward an explanation. By improving the precision with which we identify the visual content of mental imagery, a more complete appreciation of its interactive relationship with visual perception may be achieved, thereby leading to enhanced conclusions regarding the creation of cognitive representations.

Mental imagery offers a unique advantage over visual perception in that the amount of potential information available during later recall of a stimulus is far less than the amount available during perceptual viewing of the same stimulus. By definition, mental imagery refers to the ability to experience familiar or novel visual stimuli in the absence of appropriate concurrent physical stimulation (Pearson and Kosslyn, 2013). Because mental imagery is based on perceptual recall at a later time and place, it is intricately tied to memory processes. Research shows that, at a neural level, mental imagery recruits networks overlapping with visual working memory (Albers et al., 2013), suggesting that mental imagery recruits a perception-like process to recall stored information and bring it back into current consciousness for manipulation (Borst and Kosslyn, 2008; Borst et al., 2012). However, the processes do appear to be at least partially distinct, including their reliance on sensory-based visual networks as correlated with the strength of baseline mental imagery (Keogh and Pearson, 2011; see also Borst et al., 2012). Mental imagery can therefore be considered part of the output of memory, particularly in cases where a previously viewed stimulus is imagined.

Natural limitations of attention and storage capacity during the process of transduction from the sensory to perceptual level are bound to result in the loss and distortion of some visual information. In other words, the amount of information available during visual recall is both quantitatively and qualitatively diminished compared to the perceptual state. Despite this, numerous empirical studies have demonstrated that it is possible to understand, name, and describe the properties of an object through mental imagery alone (e.g., Kosslyn et al., 1995; Walker et al., 2006; Palmiero et al., 2014). Therefore, it is reasonable to infer that the reduced information available in a mental image must be at least sufficient, if not necessary, for successful object recognition. In this way, mental imagery processes may serve as a useful and natural filter by which to identify the features of an image that bear the most cognitive relevance to the viewer. By examining the output of perceptual viewing in the form of mental imagery, the number of alternative factors to be considered is greatly reduced from those present in a complex, sensation-based visual environment. Rather than attempting to measure the relative classificatory value of all available features in a given stimulus, the researcher may use the content maintained in the observer’s mental image to guide the identification of meaningful visual cues.

The goal of this review is to suggest that visual mental imagery, the contents of which have undergone a natural filtration process to sift out object information that is irrelevant for recognition or categorization in a given scenario, holds significant potential for identifying object features that are critical for perceptual recognition. These features, referred to as distinguishing or diagnostic features, are visual components with classificatory significance that facilitate fast and efficient object recognition (Baruch et al., 2014). Due to the present lack of empirical studies which directly explore diagnostic feature identification in mental imagery, this article will instead examine and discuss the practicality of such an investigation by:

(1) reviewing the prominent behavioral and neuroimaging methods that have been used to successfully access mental visual representations;
(2) evaluating the potential of each method for identifying diagnostic features based on the efficiency and specificity they have been shown to achieve;
(3) suggesting possible directions and implications for exploring distinguishing features through mental imagery in future research.

### Theoretical Background

#### Object Recognition in Visual Perception

The significance of object features in visual perception has long been acknowledged. In one of the oldest and most well-known theories emphasizing the importance of distinct visual parts, Biederman (1987) proposed that recognition of any given object depends upon an interaction between individual structural components and their overall configuration within the context of the whole. Although Biederman’s recognition-by-components (RBC) theory is based upon structural geometric shapes, further research has demonstrated that general visual features need not be limited to spatially discrete, structural parts. Features may be interpreted as any compositional unit of a visual stimulus, including contours (Loffler, 2008), colors or textures (Bramão et al., 2011a), or minimal elements of contrast, such as Gabor patches (Dong and Ren, 2015). The present article accommodates this wide variation by adopting the broad conceptualization of visual features as any “discrete components of an image that are detected independently of each other” (Pelli et al., 2006). However, because of their accessibility (in that they are both easily understood and likely to be captured in cognitive and neural measurements), the studies and implications summarized in this review are most pertinent to identifying complex shape features used for recognition. Regardless of the level of specificity at which they are characterized, any essential features must be detected and integrated within the context of surrounding information (e.g., additional object features, semantic or situational context, observer goals, object set, etc.; Pomerantz et al., 1977; Martelli et al., 2005).

Despite the extensive literature regarding the role of visual features in object perception, the precise extent to which various individual features contribute to recognition remains inconclusive. Some theories speculate that distinguishing features play a critical role in facilitating efficient object identification and categorization. These unique informative visual components expedite the determination of object identity within a specific context by allowing the observer to quickly and effectively discriminate amongst probable alternatives (Baruch et al., 2014). Similar to general visual features, the visual content of a distinguishing or diagnostic feature varies and may comprise self-contained components, such as a structural shape, or more distributed elements, such as color (Bramão et al., 2011a,b). Importantly, the properties of a distinguishing feature in any given situation vary in relation to the context of the viewing scenario (Baruch et al., 2014; Schlangen and Barenholtz, 2015), as well as external cognitive factors (e.g., selective attention; Ballesteros and Mayas, 2015).

The existence of distinguishing features in visual perception and their role in object recognition have received some empirical support, although the findings are by no means definitive. Originally thought to play an integral role in novel viewpoint recognition, diagnostic features supported a counter argument to the structural description theory suggested by Biederman (1987). Whereas the RBC theory predicted relatively stable recognition performance across novel viewpoints so long as relevant structural features, or geons, remained visible, the viewpoint variant counter argument claimed that this pattern occurred only when distinct diagnostic features were available to the viewer (Tarr et al., 1997). These informative visual components are thought to facilitate both categorization and recognition decisions by providing diagnostic discrimination amongst possible alternatives. Note here that saliency, or the degree to which a feature stands out or is noticeable or attention-grabbing, does not imply diagnosticity, which indicates utility in recognition or classificatory processes. Consider, for example, the difference between a tiger and a zebra; though the stripes are salient and highly noticeable, the mere presence of stripes alone is not enough to identify one over another. In contrast, when tasked with identifying a zebra in a crowd of cattle, stripes are more exclusionary; salience and diagnosticity overlap.

The tendency for particular features to receive increased allocation of attention, along with their potential to aid fast and efficient object recognition across dynamic scenarios, supports the plausible perceptual and cognitive significance of distinguishing features. These findings also suggest that essential distinguishing features are likely to remain relatively intact in mental representations for several reasons:

(1) their classificatory significance decreases the likelihood that they are filtered out as irrelevant information during the initial encoding of a mental representation;
(2) it is reasonable to predict that distinguishing features represent a significant proportion of semantic object information within a spatially condensed visual unit;
(3) their cognitive relevance presumably increases their robustness against the information degradation and bias effects that occur between the visual perception and mental image generation stages.

Therefore, distinguishing object features are a prime candidate for empirical study in that they are simplified, reliable units that represent or anchor the larger, more complex mental representation of a visual stimulus. To illustrate, consider the example of a hammer. Most likely, an image of a hammer comes to mind as the word is read. Are there parts or features that seem clearer than others? Consider a simple recognition experiment in which a participant is shown an image of a hammer, with the metallic hammerhead portion removed. It is unlikely that a vaguely defined wooden handle would “activate” (as reflected in any given measure of interest) one’s conception of a hammer as efficiently as the whole object, with hammerhead intact. If the manipulation were reversed, however, with the handle removed and the hammerhead retained, one would expect much greater “activation” of the abstract concept of “hammer,” along with it all of the associations that concept entails (see spreading activation theory of memory; Anderson, 1983). The ability for a subcomponent of a greater stimulus to reach the cognitive representation efficiently in the absence of some of its typical context suggests that particular features are more cognitively diagnostic than others. Once said diagnostic features are identified (say, the hammerhead in this scenario), the process of reduction – as was done by removing first the hammerhead and then the handle – can be systematically advanced to determine the smallest component, or group of components, capable of efficiently representing the cognitive concept. Thus, visual features essential to activating one’s cognitive representation of any given object may be identified. When compared across multiple exemplars and stimulus categories, any similarities in distinguishing features (e.g., shape structure, contrast, edges, etc.) may be identified. This unique ability for discrete diagnostic elements to serve as a link to more holistic or complex cognitive representations makes them a valuable subject of study, with the potential to illuminate not only the mechanisms underlying mental object imagery, but their relationship to perceptual recognition as well. The ability to reduce complex objects into their essential and most basic components could potentially lead to improved theories and methods capable of accommodating a wide range of interactions between viewing scenarios and the characteristics of natural visual stimuli – a persistent challenge currently facing traditional sensory-based perceptual research in object recognition.

It is worth noting here the extensive work done in computer vision related to the very issues addressed in this review. A recent study by Ullman et al. (2016) crowd-sourced diagnostic information of distinct features which had significant effects on human object recognition. Responses from over 14,000 human observers yielded minimal recognizable configurations (MIRCs) in 10 grayscale images depicting objects of different classes. Multiple MIRCs, each of which contained minimal redundant object information relative to the full-object image, were identified for each of the images, and allowed successful classification via limited visual regions. Their study also revealed that current computational models fall short of accurately replicating human feature-based recognition processes (e.g., model recognition for sub-MIRCs compared to MIRCs did not decrease as sharply as it did in human observers and models are unable to recognize further subordinate features within a MIRC). Ullman et al.’s (2016) representative study illustrates that it is possible to reduce complex objects to a minimally recognizable level agreed upon by a large group of observers, to the point that contribution of each feature contributes critically to recognition. Other studies performed through computer-based games show promise for incentivizing large scale data “algorithms” and computations performed by humans under the pretense of entertainment (von Ahn, 2006), and have collected data for object labels as well as locations of objects within a scene. These techniques and their potential for identifying feature-related recognition behavior in a large and diverse pool of subjects are worth keeping in mind as the review goes forward.

Although computer vision work, the details of which are beyond the scope of the current discussion (see Nixon and Aguado, 2012; Shokoufandeh et al., 2012 for recent reviews), is relevant and informative for understanding visual cognition, current computational models fail to accurately replicate human visual processes, and therefore will not be discussed in depth here. The review that follows focuses on the methods aimed at directly accessing mental imagery processes in humans in a direct and quantifiable manner, with the acknowledgment that findings from the methods discussed herein could be appropriately applied to web-based computational techniques to improve not only the accuracy of computational simulators, but the understanding of feature related perception and later mental imagery.

#### Object Representations in Mental Imagery

The long and challenging history of mental imagery research has led to at least two distinct perspectives on its relationship with visual perception. One perspective holds that neural and phenomenological processes that occur during visual perception and mental imagery are similar in function and structure due to shared underlying neural mechanisms. These commonalities extend to and include the involvement of early, retinotopically mapped visual areas such as primary visual cortex (V1; Slotnick et al., 2005; Albers et al., 2013; Pearson et al., 2015). Experiments employing transmagnetic cranial stimulation have yielded corroborating evidence of the neural overlap between perception and imagery (Cattaneo et al., 2012). Shared mechanisms have also been suggested by studies of similar reaction times in response to visually perceived and mentally generated images, robust against effects of luminance, contrast, motion, and orientation (Broggin et al., 2012). Further studies have even demonstrated that the luminance of an imagined stimulus is capable of eliciting an involuntary reaction in pupil constriction, consistent with patterns observed during perceptual viewing (Laeng and Sulutvedt, 2014). However, even those studies that report a significant amount of overlap in patterns of behavioral responses or neural activation between perception and imagery often note discrepancies in its completeness and uniformity. For instance, manipulations of spatial frequency resulted in dissimilar patterns of reaction time for real stimuli when compared to imagined (Broggin et al., 2012). Cortically, shared activation observed during imagery and perceptual processes was found to be more consistent in frontal and parietal cortical regions than in retinotopic visual areas, although significant levels of mutual activation were identified in these areas as well (Ganis et al., 2004). However, comparisons employing more flexible analytical methods such as multivariate pattern analysis (MVPA) have revealed more reliable overlap in early visual areas (Albers et al., 2013). These common neural mechanisms are also thought to underlie reported phenomenological similarities between visual perception and mental imagery. For example, there is evidence that mental images share several spatial qualities inherent to objects perceived in the visual field (Kosslyn et al., 1983), as indicated by observed effects of mental spatial rotation, mental scanning, and time required for size-inspection tasks (D’Ercole et al., 2010). Structural theories of mental imagery further propose that, similar to perceptual stimuli, imagined images support a limited resolution and defined sense of spatial extent (see Finke, 1985 for review), including spatially equivalent individual units (Kosslyn et al., 1983) and overall similar visual content (Nanay, 2014).

The nature and extent of shared neural foundations underlying perceptual and imaginative processes is called into question by several unique clinical cases. Vivid mental imagery has been identified in at least one patient exhibiting highly localized cortical damage to V1, resulting in severe deficits in perceptual task performance (Bridge et al., 2011). Neural recordings collected through functional magnetic resonance imaging (fMRI) further revealed that the patient’s activation patterns during mental imagery episodes were similar to those of healthy sighted subjects; behavioral testing confirmed these results. Additional assessments indicated greatly attenuated perceptual abilities, suggesting that visual mental imagery remained intact in the absence of healthy early visual cortical networks. Patients experiencing visual agnosia (Behrmann et al., 1994; Servos and Goodale, 1995) and those with congenital ocular blindness or lifelong visual impairment have also been found to retain visual imagery capabilities, although performance deficits vary in regards to the nature of the impairment in the latter (see Cattaneo et al., 2008 for review). A recently recognized neuropsychological disorder exhibits the opposite pattern. Aphantasia is characterized by the inability to produce visual mental images while perceptual object recognition performance remains unimpaired (Bartolomeo, 2008; Zeman et al., 2015). This intriguing condition has been documented in several otherwise healthy individuals who report a sudden loss of the ability to generate forms, shapes, and colors in their mind’s eye (Bartolomeo, 2008; Moro et al., 2008; Zeman et al., 2010). Although cortical lesion (Zeman et al., 2010), congenital (Zeman et al., 2015), and psychogenic (de Vito and Bartolomeo, 2015) origins are generally suspected, the disorder is poorly understood at this time. Nevertheless, the precise double dissociation offered by these unique clinical cases suggests that the neural correlates of perceptual recognition and mental imagery are at least partially distinct. However, the inferences drawn from these studies are limited due to the unpredictable nature of cortical injuries and their effects on cognitive functioning, and the origins and effects of aphantasia have only just begun to receive careful empirical assessment.

Taken together, the existing literature varies considerably regarding the neural nature of object representations in mental imagery. The lack of compelling support for any one theory over another favors an agnostic stance on the precise nature of mental representations and their neural underpinnings. This review includes articles regardless of where along the spectrum of theories their conclusions align and refrains from making judgments on the validity or accuracy of findings based solely on theoretical perspective.

#### Diagnostic Features in Mental Imagery

Although distinguishing features have yet to be directly identified in mental imagery, related research does support their existence in this modality. Several fMRI studies have successfully predicted category classification of an imagined stimulus by applying computational analyses such as pattern classifiers (Reddy et al., 2010) and voxel-wise encoding models of tuning to low-level visual features during viewing tasks (Naselaris et al., 2015). Considering the important role distinguishing features are believed to play in perceptual categorization tasks (Baruch et al., 2014), it is conceivable that highly diagnostic visual components are primary contributors to this type of neural decoding. However, the most informative locations from which decoding decisions may be achieved vary across studies. Activation patterns within the ventral-temporal cortex were found to be more reliable for imagery categorization decoding than those within early retinotopic cortical regions (Reddy et al., 2010). Another study reported evidence that low-level visual features of mental imagery for remembered scenes are encoded in early visual areas (Naselaris et al., 2015). Behavioral data such as response time and error rate indicate that it is indeed possible to extract low-level partial features, such as T junctions, from holistic mental representations with the same proficiency as perceptual evaluations, although high-level properties, including global symmetry, are more easily evaluated in both conditions (Rouw et al., 1997). Together, these empirical findings add substance to long-standing theories suggesting that component units are available within holistic mental imagery (Kosslyn et al., 1983).

Despite the inconsistent neural regions reported by decoding studies, the ability to predict category information from neural activity recordings at all holds important implications for revealing diagnostic feature information in mental imagery:

(1) because diagnostic features facilitate efficient perceptual category classification via uniquely discriminating visual features, neural activity that supports category classification may be related to diagnostic feature information, thus indicating that component-based visual information is directly represented through neural substrates;
(2) because the exact locations within the neural visual processing stream at which distinguishing features are represented remains unclear, it can be assumed that diagnostic feature content may exist in either high or low visual areas.

## Exploration and Evaluation of Methods

### Behavioral Methods

#### Questionnaires

Despite mental imagery being a difficult and abstract concept to target, several self-report questionnaire instruments have demonstrated successful and reliable measurement of various aspects of visual representations. Of particular note are the widely used Vividness of Visual Mental Imagery Questionnaire (VVIQ; Marks, 1973) and its more recent revisions, the Vividness of Visual Mental Imagery Questionnaire-2 (VVIQ-2; Marks, 1995) and the Vividness of Visual Mental Imagery Questionnaire-Revised Version (VVIQ-RV; Marks, 1995; Campos, 2011). In each of these surveys, participants are prompted to visualize specific scenes, such as a sunset, and report on the clarity and detail of the generated images using Likert scale responses. The variants of the VVIQ differ on whether they require the participant to visualize with eyes open or closed. Critical statistical testing of the original VVIQ and both of its variants indicate high internal validity for measuring the mental imagery construct (Campos, 2011). In addition, the Plymouth Sensory Imagery Questionnaire (Psi-Q) is a unique assessment able to provide highly reliable measures of individual tendency to experience vivid imagery across multiple modalities (Andrade et al., 2014). The demonstrated internal validity of assessment items which require participants to generate detailed scenes indicates that individuals are able to perceive multiple and specific visual components in mental representations, and that these components can be reliably captured by straightforward survey items.

At least one questionnaire instrument has attempted to target specific shape information represented within visual mental images. The Mental Imagery Scale (MIS; D’Ercole et al., 2010) was designed to exploit the relationship between verbal descriptions and mental images in order to directly translate structural features present within mental representations into precise verbal descriptions. As the creators note, this type of scale is advantageous for highly visual and communicative fields such as architecture and art didactics. To test the MIS, participants were given a verbal description of a piece of artwork and asked to answer questions aligned with one of six factors describing aspects of mental images and the process of image formation: Image Formation Speed, Stability, Dimensions, Level of Detail, Distance, and Perspective (D’Ercole et al., 2010). The results of the study showed that participant responses supported the proposed six factor model, suggesting that mental imagery is influenced by inherent spatial properties. As it relates to the study of diagnostic features, this instrument demonstrates that reliable and detailed assessment of visual mental imagery is achievable through verbal descriptions alone. If this specificity were to be increased to the level of independent, discrete object components, it is possible that the MIS or similar instruments could target and identify discrete, classificatory visual features through self-report.

The Object-Spatial Imagers Questionnaire (OSIQ; Blajenkova et al., 2006) approaches the level of specificity necessary to identify distinguishing features by assessing object imagery preferences at the level of the individual. However, the purpose of the OSIQ is to reveal individual tendencies for representing images in a holistic, picture-like fashion or spatially, through a compilation of individual parts; the questionnaire does not include explicit appraisal of shape. Tests of the OSIQ demonstrate varying levels of preference for holistic and part-based representation across individuals. These results hold important implications for any study investigating mental imagery, because individual preference for holistic representations could lead to increased Type II error rates when attempting to access part-based visual information. In comparison to the OSIQ, the VVIQ has not been shown to characterize these spatial preferences (Blajenkova et al., 2006), which may be a result of the VVIQ’s focus on contextual visual scene imagery, as opposed to independent objects. Regardless, it would be prudent for future studies to consider the possibility of individual differences in representational style when choosing a questionnaire measure, as well as when analyzing and interpreting study findings.

There are several advantages and drawbacks to employing questionnaires in the study of mental imagery. On the one hand, surveys allow a large amount of detailed data to be collected in a relatively short amount of time, much more so than physiological or biological measures; the questionnaires described above contain 32 items on average. All items consist of a simple Likert scale rating ranging from 5 to 7 steps. In addition, these measures require very little in the way of technical skills or eligibility criteria, making the instruments accessible to a broad and representative population. The reliability of self-report responses of this type is also supported by behavioral results indicating that individuals tend to have reliable and accurate metacognition of their own imaginative experiences (Pearson et al., 2011). However, several complications arise when an individual is asked to verbally describe or physically recreate visual content. For example, perceptual biases and lack of artistic ability may distort participants’ drawings of images, and verbal descriptions may be misinterpreted or incomplete. Indeed, studies of drawings by non-artists have shown that drawing errors are positively correlated with perceptual biases encoded during initial image observation (Ostrofsky et al., 2015). Most importantly, the very nature of questionnaires makes the probing of specific distinguishing features difficult to accomplish without introducing artificial bias. Furthermore, even when bias is minimized, responses are likely to capture only those spatially discrete shapes which lend themselves to canonical lexical labeling.

Despite these shortcomings, the high level of proficiency with which written questionnaires have been shown to access the mental imagery construct warrants their consideration as reflectors of distinguishing features in mental imagery. In order to best take advantage of the benefits provided by their time-efficient and portable format, questionnaires assessing the specific shape structure of visualized images may best be applied to a large group of respondents. Using an extensive population reduces the influence of individual biases and representational preferences on responses. Any significant patterns observed within and across responses could then be identified and targeted for further, more in-depth, analyses. In the meantime, indicators of individual preferences such as the Psi-Q and OSIQ should be considered for use as covariates when measuring partial object information in mental imagery, regardless of the primary methodology employed. Even perceptual biases revealed through drawings may be insightful for inferring the visual aspects which receive the most attention during encoding, thus suggesting features of greater relative cognitive import. If diagnostic features are highly informative for the identity of a given object, patterns among the features or shape aspects reported by a large and varied group hold potential for identifying naturally occurring diagnostic object features. Although the distinguishing features captured by questionnaires would most likely be limited to spatially discrete, nameable object components, these data could then be used to guide further empirical research to evaluate the quality, reliability, and validity of these components as perceptual diagnostic features.

#### Motor Behavior

Gestural motor movements have also been explored as an indicator of mental object representation content. Following an established link between functional motor actions and tool use, one such study investigated whether an individual could acquire functional object representations merely by imagining the use of novel objects and visualizing the appropriate corresponding hand gestures (Paulus et al., 2012). Participants were shown pictures of four artificially designed objects with unique functional ends that required distinctive hand grips in order to be brought toward the ear or the nose. Prior to training, participants were instructed on the proper action associated with each object and told to imagine a salient effect resulting from that action (e.g., smelling an odor or hearing a sound). Each participant was trained on two of the four novel objects over three training blocks interspersed between three alternating test blocks. Training blocks consisted of a stimulus image displayed on a screen, followed by the presentation of a photograph in which an actor depicted the object at its correct final action location. Object representations were assessed in subsequent test trials, during which participants were asked to indicate with a button-press whether an object shown in an action demonstration matched the object image that was displayed immediately prior. The results of the study revealed slower response reaction times to images in which a trained object was depicted at an incorrect end location as opposed to a correct one. However, this response time did not vary as a function of whether or not the object in the action demonstration was held using a correct or incorrect grip (Paulus et al., 2012). The sensitivity to action-related end location suggested by response time patterns indicates that participants successfully acquired object representations which included information regarding typical end goal location. The authors of the study propose that proper grip was not encoded in object representations as strongly as motor action due to the fact that participants were instructed only to visualize a salient effect resulting from grip manipulation and never received physical, concrete experience in this aspect. However, the researchers note that this effect may also be related to the novelty of the objects included in their study, and they predict that grip may be more relevant and revealing of object representations when associated with stimuli with which participants have had previous experience.

The findings yielded by the study performed by Paulus et al. (2012) serve to illustrate the importance of the goal end of an object as a key feature of functional object representations. Because motor planning requires an understanding of the object to be interacted with, which in some cases is completely determined by a unique functional end, it is highly likely that motor imagery is related to the type of visual mental imagery performed during object recognition. The interaction between visual cognition and efficient motor planning has been observed in both adults (Janczyk and Kunde, 2012) and infants (Barrett et al., 2008). Although motor planning is thought to be analytical in comparison to object perception, which is argued to generally rely on combined features (Janczyk and Kunde, 2012), this may favor motor planning as a more accessible pathway by which to identify individual features important for visually driven behavior. Paulus et al. (2012) study adds further support to the relative diagnosticity (in this case, diagnostic for classifying the appropriate grasp or movement) of particular object features over others and also suggests a potential avenue for identifying integral object components through associated motor behaviors. Previous research suggests that goal ends of objects are likely to carry categorical information related to their uses and the means, or action behaviors, by which those uses are efficiently achieved (e.g., Creem and Proffitt, 2001). Numerous studies of motor imagery explored through near-infrared technology further illuminate these findings; these are discussed in Section “Neural Activity.”

The implicit connection between gestural actions and the cognitive understanding of objects holds intriguing potential for the study of distinguishing features, but it is subject to significant weaknesses as well. Similar to questionnaires, motor behavior tasks provide a non-invasive, inexpensive method by which to assess distinguishing object parts that inform natural interactive behaviors. However, such testing is considerably more time-consuming than survey administration, and the resulting data require complex scoring and careful interpretation. In order to avoid confounds of novelty and inexperience, investigations of motor behavior regarding distinguishing features may best be applied to ecologically valid objects with which participants have had previous physical interactions. Categorical classification as implicated by specific gestures may allow for efficient object decoding based upon observation alone (Rosenbaum et al., 1992). However, this type of gestural relationship is acutely limited to manipulable objects and, what is more, manipulable objects that are associated with a clearly recognizable, stereotypical gesture. Nevertheless, implicit assessment of object features or categories through functional motor movements may illuminate the spatial locations and qualities of features that are typically targeted in motor movements. Based on the established functional connection between motor actions, such as grip, and the end location of an object (Rosenbaum et al., 1992), motor behavior therefore holds the potential to indicate essential structural features in tools and other manipulable objects. This method may be combined with data collected from other techniques used to assess diagnostic object features, such as questionnaires or neurophysiological measures, in order to form a more complete understanding of an object mental representation and its cognitively informative distinguishing features.

#### Eye Tracking

Eye movements associated with imaginary visual tasks are similar to those observed during perceptual tasks. Spontaneous eye movements during visualization of a scene reflect directional patterns comparable to those associated with perceptual viewing (Laeng and Teodorescu, 2002). Participants report experiencing increased difficulty in producing visual mental images when instructed to restrict their eye movements while doing so. When visualizing under this constraint, participants’ descriptions of the imaginary scene tend to become less detailed and limited to rudimentary features (Laeng and Teodorescu, 2002). The enhanced difficulty with which detailed visual mental imagery is produced when eye movements are restricted signifies an automatic, perhaps interdependent relationship between eye movements and the processing of visual imaginary scenes.

The prediction of an association between mental imagery content and concurrent oculomotor movements is by no means a novel one, and it has received empirical support dating back several decades (Brandt and Stark, 1997; Spivey and Geng, 2001; Laeng and Teodorescu, 2002; Johansson et al., 2006; Holm and Mäntylä, 2007; Ryan et al., 2007; Hannula and Ranganath, 2009; Williams and Woodman, 2010; Johansson and Johansson, 2014; Martarelli et al., 2016). In a direct comparison between visual inspection and mental visualization, repetitive sequences of fixation across diagrammatic checkerboard stimuli were recorded and analyzed in relation to the scanpaths observed during mental imagery of the same stimuli (Brandt and Stark, 1997). Participants were first familiarized with a checkerboard stimulus for 20 s and subsequently prompted to visualize the pattern on an empty grid for 10 s, followed by a second viewing period of 10 s. The protocol was repeated three times; stimuli were rotated by 90° in each subsequent trial, and eye movements were recorded using a video-based eye monitoring apparatus. String editing analysis of observed scanpaths across the two conditions revealed a high degree of similarity in saccadic patterns, suggesting that eye movements may play a role in organizing the visual content of a mental representation in the absence of physical stimuli. Although indications of grid size and location remained relatively consistent, scanpaths observed during imagery trials were found to be about 20% smaller than those observed during viewing trials, indicating an analogous but not identical relationship between saccades and the representations they reflect (Brandt and Stark, 1997), perhaps stemming from disparities between the representations and their physical counterparts. Nevertheless, the parallels observed in oculomotor patterns in this experiment lend strong support to the employment of eye movement behavior as an index of object features.

Although the precise nature of the relationship between saccades and object perception is still debated, there is some evidence that saccades index attention to specific object features during visual search. Eye tracking data suggest that saccadic patterns are influenced by peripheral object information acquired during visual search, thus reflecting attention to particular visual features based on available object information (Herwig and Schneider, 2014). Early fixations are also drawn by objects that retain intact low-level visual properties but are altered to exhibit object-intrinsic anomalies, such as unnatural rotation or color distribution, implicating the influence of peripheral object analysis on saccadic eye movement (Becker et al., 2007). These findings lend credence to the possibility that saccades index relevant, object-specific features based upon the observer’s pre-saccadic processing of the image.

There are several limitations that must be considered when applying eye movement tracking to the study of object feature detection, both in perceptual and imaginary tasks. The first of these is the potential confound of covert attention, during which an observer allocates increased cognitive attentional resources to a particular location in the visual field without executing a saccadic eye movement (Mccarley et al., 2002). The ability to manipulate attention in the absence of a change in physical behavior further reduces the reliability of eye movements as a direct and reliable indicator of active cognitive processing. Studies revealing poor memory performance despite accurate saccades to the location of previously displayed stimuli suggest that object properties are not necessarily coded in conjunction with spatial location (Richardson and Spivey, 2000; Johansson and Johansson, 2014). Similarly, tests involving eye movement manipulations during mental imagery have shown greater adverse effects on spatial aspects of mental imagery than on visual details (de Vito et al., 2014). Lack of spatial sensitivity and precision both in eye tracking equipment and the human fovea contribute to these issues.

Nonetheless, the relationship between oculomotor movements and spatial locations may be used to the advantage of object feature research. If discrete object features were equated to independent, distinct spatial locations, similar to the design employed by Brandt and Stark (1997), this connection could provide an opportunity to index individual feature attention through eye tracking. By equating discrete visual components with unique locations outside of the foveal visual field, participants are more likely to execute oculomotor movements in order to fixate individual visual features, thereby increasing the spatial resolution with which specific distinct features may be identified. The order, frequency, or duration of fixations on particular units may suggest a feature that is more salient than others, and may then be tested for efficiency in categorization to determine diagnosticity. This type of investigation could be applied to visual object search and subsequently compared to an analogous mental imagery condition. Several issues remain to be resolved before attempting such an experiment with real-world object stimuli, including the decision of the appropriate size at which object parts should be delineated, thus manipulating the amount of overall object information each unit comprises. In addition, changing the size of an object can alter one’s perception of it Sterzer and Rees (2006), and modifying the spatial configuration of an image may have deleterious effects on its holistic properties, thus influencing the manner in which it is processed (e.g., Martelli et al., 2005). Because the goal of object recognition research is to access the natural perception of stimuli and to identify the properties that facilitate this perception, it is important to minimize the amount of bias introduced by experimental manipulation. These concerns must be carefully addressed if confident inferences are to be made from the association between object features and spatial locations, but the benefits for understanding attention to classificatory visual components could be substantial.

### Neural Activity

#### Functional Magnetic Resonance Imaging

A large number of neuroimaging studies suggest that distinguishing object information is represented at the neural level and can therefore be detected by brain recording equipment. Data collected from fMRI have been used to successfully decode both object identity and category classification of not only visually perceived stimuli, but mentally generated images as well (Thirion et al., 2006; Reddy et al., 2010). Perceptually, low-level visual features as precise as edge orientation have been decoded from neural activity and utilized to reliably classify which of a small set of stimulus orientations were being viewed by a participant (Kamitani and Tong, 2005). A study involving mental imagery of 60 object line drawings, each belonging to one of 12 categories, found that each category was marked by a similar distribution of activated voxels which remained stable across categories and subjects, particularly in temporal, occipital, and fusiform gyrus cortical regions (Behroozi and Daliri, 2014). The consistency in voxel activation observed across individuals suggests that the recorded neural responses were driven by some inherent property or features of the stimulus itself which signify its membership to a particular category, thus reducing the likelihood that individual differences or bias factors influenced the pattern of neural response. Other studies have found similarly distinct results in the absence of visual stimulation, such as the dissociation of imagined face and place stimuli based on corresponding stimulus-specific cortical regions (O’Craven and Kanwisher, 2000), classification of imagined object categories as reflected in ventral temporal cortex (Reddy et al., 2010), recreation of simple checkerboard stimuli based on activation found in early retinotopic regions (Thirion et al., 2006), and even decoding the category and identity of dream content (Horikawa et al., 2013). At least some studies of mental imagery involving imagination of simple stimuli have achieved limited success in this area through the use of MVPA (Reddy et al., 2010; Albers et al., 2013; Behroozi and Daliri, 2014). These results suggest that information related to the category and identity of imagined stimuli is reflected in neural activity in a manner similar to that during viewing and which is accessible with existing technologies, though it may be more challenging to differentiate than that of perceptual activity.

The impressive precision and flexibility with which fMRI data have been shown to capture specific feature information support the possibility that distinguishing object components are reflected in neural activity associated with mental imagery, thereby providing a direct means of accessing diagnostic features through mental representations. If distinguishing features truly are sufficient for category classification, as theory suggests, they may be significant contributors to the neural patterns observed in studies such as those described above. In order to test this, categorical stimuli that are found to elicit similar patterns of neural activation could be systematically segmented into a collection of visual component parts (similar to Ullman et al., 2016). These parts may then be presented individually during fMRI to identify which, if any, featural units are capable of eliciting a neural response similar to the one associated with the original, intact object. Although this method of segmentation would preclude cases in which holistic information serves as a diagnostic feature, so long as perceptual comparisons are restricted to the group level and not the individual level, the distinguishing visual features useful for recognition in this type of task should be shared across multiple exemplars. This is because global information comprises a specific configuration of multiple features and is therefore less useful for efficient categorization across several objects, some of which may not share all of the characteristics contained in the holistic representation.

#### Electroencephalography

In addition to fMRI, electroencephalography (EEG) has been used to study mental representations as expressed through electrical neural activity (e.g., Shourie et al., 2014). Research in object-related mental imagery using EEG is comparatively scarce, but the work that has been done has been successfully used to decode motor imagery for the purpose of brain–computer interface control (e.g., Townsend et al., 2004; see Choi, 2013 for review), suggesting EEG is capable of distinguishing amongst general categories of imagined actions. In a marked and significant departure from the general trend to focus on holistic information within mental imagery, one study attempted to investigate the role of partial object information through changes observed in the EEG spectrum (Li et al., 2010). Participants were shown gray line drawings of 60 common objects containing what the researchers defined as distinct, nameable, spatially discrete features. During the experimental task, EEG data were collected while participants were presented with a line drawing stimulus for 500 ms. Following a 4000 ms pause, participants were cued to generate a mental image of the previously viewed line drawing according to the displayed holistic or partial-image word cue. These lexical cues referenced either the canonical name of an entire object or the name of one of its semantically meaningful parts (e.g., the word “lamp” cued a holistic imagery condition, whereas “lamp shade” prompted imagery for only a specific region of the stimulus; Li et al., 2010). A button press on a response box indicated the starting time point for each imaginary episode. The results of the study revealed that the greatest differences between perceptual and imaginary tasks existed within the theta and alpha band spectra. Although both conditions elicited responses significantly above threshold, partial imagery showed an earlier “burst time” and lower alpha power than the holistic condition, and coherence differences were observed in frontal and central-temporal electrode regions (Li et al., 2010). The authors speculate that the early time onset associated with the partial imagery condition indicates that partial visual information manifests independently from holistic imagery generation, and that stronger theta power in this condition reflects more complex processing required to retrieve object details. However, the concurrent decrease in alpha band energy in the partial imagery task is proposed to reflect a “creative or modifying process” that is not required for simple memory retrieval of holistic images (Li et al., 2010). These findings suggest that, despite its apparent independence from holistic information, partial imagery formation appears to involve complex interactions with its associated holistic context. This could have important implications for understanding the relationship between distinct diagnostic features and the whole representation to which they are related. Similar to Ullman et al. (2016), these results also suggest that distinguishing features may be embedded within a larger configuration, or a diagnostic feature may actually comprise a collection of individual features. This is an important consideration when attempting to identify minimally diagnostic regions, but Li et al.’s (2010) study suggests that EEG may be sensitive to this process.

The word cue approach employed to manipulate the experimental mental imagery condition in Li et al. (2010) remains to be validated as a reliable method for inciting holistic and part-based images and is a primary concern when interpreting the results of the study. The use of word cues based on nameable object parts forces an artificial separation of an image into spatially discrete features defined by recognizable, but arbitrary, structural features, the interactive relationship between each and the overall holistic representation is unclear. However, there is evidence that verbal cues are capable of evoking mental imagery with some level of precision, as demonstrated by written survey instruments such as the MIS (D’Ercole et al., 2010), and that the category of the mental image may be discernible in EEG data (Simanova et al., 2010). In addition, a computational model used to decode human brain activity to predict fMRI activation associated with the meaning of nouns (Mitchell et al., 2008) indicates that word cues can modify neural and, by extension, cognitive activity. Therefore, although the object part manipulation applied in Li et al. (2010) warrants scrutiny, the study’s finding that patterns in EEG spectrum activity were able to discriminate some level of variation in mental representations remains worthwhile for consideration in the exploration of partial features in mental imagery.

#### Functional Near-Infrared Spectroscopy

Functional near-infrared spectroscopy (fNIRS) is a relatively new technology that is rapidly gaining popularity due to its portability and flexibility of experimental application. This system combines the spatial sensitivity of fMRI with the convenience and temporal resolution of EEG through non-invasive measurement of the diffusion rate of near-infrared light as it is projected through the skull. Recordings collected from fNIRS are influenced by the relative concentration of oxygenated and deoxygenated hemoglobin in cortical blood flow, and are thus conceptualized as an indirect measurement of neural activity (Kamran and Hong, 2013). This comparatively recent methodology has yet to be directly applied to tasks involving visual mental imagery generation. However, studies of infant perception, as well as a substantial base of motor imagery decoding literature, show mixed evidence for hemodynamic responses recorded via fNIRS as an index of private mental and visual processes.

A study of visual processing in infants employed fNIRS in an investigation of the neural correlates underlying object individuation (Wilcox et al., 2005). Using a version of the narrow-screen task (Wilcox and Baillargeon, 1998), infants were familiarized to two featurally distinct objects, a ball and a box, as they successively appeared from opposite sides of a narrow or wide screen. Behavioral response times revealed that infants looked longer at the narrow screen condition, suggesting that the infants were able to discriminate the stimuli as two distinct objects which could not logically fit behind the screen along the same axis simultaneously. Variations in hemodynamic response as measured by NIRS during narrow screen trials were localized to the primary visual and inferior temporal cortex, indicating that object individuation is associated with unique, detectable patterns of neural activity in these regions (Wilcox et al., 2005). Although the researchers acknowledge that more work remains to be done before the connection between variations in oxygenated and deoxygenated hemoglobin and cognition are well-understood (but see Chen et al., 2015), their study does support fNIRS as a viable means of indexing private visual phenomena related to object recognition processes. Furthermore, the capacity for fNIRS to discriminate amongst neural activity influenced by variations in distinct local features shows promise for detecting distinguishing features in object recognition.

Several concerns remain when considering fNIRS as a measure of mental imagery. On the one hand, a relatively established body of work investigating motor imagery may hold clues for guiding future application of this technology to object representations. The bulk of this literature focuses on decoding motor imagery for application to brain-computer interface technology (see Naseer and Hong, 2015, for review). Accordingly, NIRS measurements are frequently recorded from regions of motor cortex, which tend to be easily penetrated by near-infrared. Although experiments that apply fNIRS to motor imagery decoding do not attempt to access visual mental imagery directly, their results demonstrate the potential for fNIRS data to facilitate a reliable decoding of private internal events. This may indicate that the same methods could be applied to visual object mental imagery, so long as relevant cortical surfaces can be reached (to be discussed later in this section). If the neural substrates of mental imagery can indeed be accessed, the high temporal and spatial resolution afforded by fNIRS technology may be a worthwhile avenue through which to pursue diagnostic features represented in mental imagery. Unfortunately, skepticism on the accuracy and utility of fNIRS recordings, even in motor imagery, remains (e.g., Waldert et al., 2012), which prevents confident recommendation of the application of fNIRS technology, in its current state, to object imagery decoding.

#### Summary of Neural Activity Measures

The investigation of mental imagery through brain activity is clearly advantageous in that these methods do not require individuals to explicitly communicate their private mental experiences. Despite the demonstrated success of neurophysiological recording methods in accessing mental imagery, there are still several limitations that must be considered when examining their implications for identifying distinguishing features in object representations. For example, similar to the stimulus set utilized by Behroozi and Daliri (2014), successful decoding of category or identity information in fMRI often relies on a collection of images from which to decode responses, with few exceptions (e.g., Thirion et al., 2006). This requirement restricts the flexibility with which neural imaging methods can index natural imagery generation in real-world scenarios, which contain multiple variables and a huge set of possibilities for visual stimuli. However, it should be noted that some studies have managed to push the boundaries of this set to impressive limits and still report success in decoding components of mental imagery (e.g., Miyawaki et al., 2008; Horikawa et al., 2013). In addition to fMRI, EEG dynamics are likely capable of capturing effects caused by the processing of spatially defined object components (Li et al., 2010). However, as mentioned above, manipulating the spatial resolution of defined features via verbal cues poses several challenges, and, as of yet, EEG has demonstrated success mostly at the level of general or superordinate classification. The issue of avoiding artificial biases introduced by arbitrary delineation of structural features extends to any perceptual assessment of mental imagery. Careful and creative experimental designs are required in order to achieve a method capable of accessing discrete diagnostic visual features as they occur naturally and at a level accessible by EEG.

Although results from fNIRS studies show limited ability to categorize motor imagery and visual perceptual processes, there are several concerns that affect this area of research as applied to feature detection in object mental imagery. First, the nature of infrared light penetration employed by fNIRS restricts recordings to areas of cortex lying closely beneath the skull approximately 2–3 cm below the cortical surface (Wilcox et al., 2005). Fortunately, there is evidence that cortical visual processing areas are accessible via near-infrared. In addition to recordings obtained from primary and ventral visual cortex in infants (Meek et al., 1998; Wilcox et al., 2005), fNIRS has been shown to successfully index hemodynamic changes in adult human visual cortex during perceptual tasks (Takahashi et al., 2000). Furthermore, fNIRS recordings collected from adult primary visual cortex and supplementary neurophysiological measures show that fNIRS is capable of revealing patterns of stimulus selectivity as well as area specificity (Chen et al., 2015). These studies show potential for the application of fNIRS and fNIRS-hybrid methodologies to visual feature representation in adult mental imagery. However, considering the multiple findings which implicate areas outside of early visual cortex as significant contributors to visual mental images (see Vetter et al., 2014), there remains further doubt as to whether or not fNIRS can be used to reliably and thoroughly examine the neural correlates of discrete features in mental representations.

An additional advantage of neuroimaging methods is that their data lend themselves to a wide range of statistical analyses which allow interpretations of complex activation patterns to identify correlations between visual and semantic information. Many of these techniques, including MVPA and support vector machine learning, allow multiple factors to be considered when correlating brain activity with category information (see De Martino et al., 2008; Kriegeskorte, 2011; Chen et al., 2014; Haxby et al., 2014 for reviews). Other statistical techniques, such as Naïve Bayes modeling, have yielded reliable predictions of semantic category classification for pictures and words (Behroozi and Daliri, 2014). The variety of statistical methods which may be applied to neuroimaging data enhances their potential for drawing inferences between neural activity and semantic object recognition processes, which may eventually allow specific predictions to be made regarding object feature information within mental images.

Overall, fMRI neural recordings of perceptual and mental visual processing hold great potential for indexing distinguishing features in object representations, whereas EEG and fNIRS appear to be weaker methods. The sensitivity to object content demonstrated by fMRI data clearly suggests their utility for accessing the discrete visual elements necessary for distinguishing amongst different object categories. Results from EEG reflect partial imagery effects in brain wave dynamics, but current work is limited to general and disparate effects in holistic vs. non-holistic effects. In addition, there is reason to believe that fNIRS technology may be capable of recording mental imagery as expressed within adult primary visual cortical regions. However, more work remains to be done before fNIRS can be confidently applied to imaginary representations in either the motor or object imagery domains. Considering that fNIRS and EEG are highly compatible and improve accuracy when used together to investigate perceptual processes (e.g., Putze et al., 2014), combining the spatial sensitivity and broad range of fMRI with the temporal resolution of EEG or fNIRS may improve upon the weaknesses of each improve their success in accessing diagnostic features prevalent during visual imagery.

## Implications and Conclusion

The aim of this review is twofold: to propose that mental imagery is an advantageous and valid method for assessing diagnostic object features, and to demonstrate that, in spite of the present lack of direct investigations of diagnostic features within mental imagery, evidence of their relationship and the tools best suited to their study are suggested by existing literature. Each measurement method offers its own unique advantages and weaknesses for exploring the role of diagnostic visual components in object processing (see **Table 1**). The early, underdeveloped state of this field favors a systematic methodological approach capable of capturing a broad range of information. In order to achieve this, measurement approaches should be combined with the aim of capitalizing on methodological strengths and compensating for weaknesses, with an emphasis for collecting large and varied amounts of data. Here, overall implications and the utility of each method for exploring distinguishing features in visual imagery are summarized and suggestions are made for future directions.

**Table 1:** Methodological pros and cons for accessing object mental imagery content.

Method	Pros	Cons
Questionnaires	• Reliable self-report • Large respondent pool • Rapid data collection • Easily combined with other measures	• Requires verbalization of imagery experiences • Indirect access to mental imagery content
Motor imagery	• Produced spontaneously • Easily accessible via multiple measures (behavioral, EEG, possibly fNIRS)	• Limited to distinct manipulable objects • Useful for identifying functional ends, but not flexibly diagnostic features • Best applied to familiar, real-world objects with real-world practice
Eye tracking	• Produced spontaneously • Limited specificity of distinct features	• Low spatial resolution Saccades influenced by spatial location as well as feature content • Confounds of covert attention
fMRI	• Produced spontaneously • High spatial resolution • Reliable decoding of perception and mental imagery • Whole brain recordings	• Low temporal resolution • Decoding mental imagery may require advanced statistical methods • Machine learning may require large sets of training images
EEG	• Produced spontaneously • High temporal resolution • Limited success in holistic vs. non-holistic imagery classification • Whole brain recordings	• Low spatial resolution • Limited to general representational processes
fNIRS	• Produced spontaneously • Moderate temporal and spatial resolution • Limited success in motor imagery decoding	• Not previously applied to mental object imagery • Remains to be validated as reliable indicator of motor imagery • Limited access to visual cortical areas

Written survey instruments such as the Vividness of Visual Mental Imagery Questionnaire, MIS (Marks, 1973, 1995), and Object-Spatial Imagers Questionnaire (Blajenkova et al., 2006) are beneficial for collecting large amounts of detailed and moderately reliable self-report data. The fundamental methodological issues associated with asking individuals to recreate or verbalize their internal experiences limit the possibilities for applying this tool as a direct individual assessment of diagnostic features. Pooling responses over a large group provides the best opportunity to reveal meaningful trends regarding the nature of classificatory object features. In addition, the ease of use and rapid administration of self-report instruments facilitate their combination with other forms of measurement. The consideration of questionnaire responses alongside neurophysiological data collected from methods such as eye tracking, EEG, and fMRI provides several benefits:

(1) it facilitates rapid growth of the relatively undeveloped area of semantically labeled visual features;
(2) it allows important individual differences in imagery generation style to be identified and considered when interpreting supplementary, indirect measurements;
(3) it lends insight into the cognitive processes revealed by self-report, which may provide informative, concrete links between semantic classification and biological activity, thereby augmenting interpretations drawn from physiological or neurological recordings.

Studies of motor movement behavior implicate a significant relationship between goal-directed actions and mental representations that is mediated by the functional end of manipulable objects. When acquired without physical practice, however, this relationship appears to be limited to large-scale, goal-directed behaviors that are expected to produce an appropriate consequence. Because precise motor behaviors such as grip have not been shown to share a direct correlation with imagery-acquired mental representations, this approach may only appropriate for items that are associated with obvious and unique goal-directed movements, such as tools. Nevertheless, the observed correlation between spatially discrete, structural object components and motor behavior supports the likelihood that distinct features exist in mental representations, and that cognitively significant features may be indirectly expressed through physical actions.

Eye tracking findings indicate that saccades are similar across perceptual viewing and mental imagery states, and thus reflect meaningful cognitive processes. Although saccadic eye movements in either condition may implicate attention to spatial location more so than individual features, this relationship may be exploited for the benefit of diagnostic feature research. If object features were to be reliably equated with distinct spatial locations in a manner that does not artificially modify the holistic representation of an object, saccadic eye movements may allow a more direct index of attention to salient object features rather than spatial location, which can then be tested for classificatory utility. In addition, such a method may pre-empt possible confounds of covert attention by requiring overt eye movements in order to fixate discrete features. This approach could be applied to perceptual object recognition tasks, the results of which could guide and be compared to analogous mental imagery investigations. A straightforward assessment of diagnostic features across perceptual and imagery conditions such as this may lead to further insight into the broader interactions between object features, perceptual processing, and mental imagery.

Neural activity recordings obtained through fMRI constitute the most established area of research regarding internal visual images. This method provides a direct index of mental imagery content while avoiding the complications associated with verbal or visual translation of private mental experiences. Category classifications achieved via blood-oxygen-level dependent fluctuations as measured by fMRI strongly implicate the presence of diagnostic feature information in mental images, as well as their expression through neural activity. Distinct patterns of activity associated with particular categories of imagined objects have been identified and found to remain stable across individuals. Overall, fMRI techniques show notable promise for advancing the understanding of the role of diagnostic object features in object representations via the underlying neural mechanisms of mental imagery. Preliminary findings from EEG studies also suggest that holistic and non-holistic imagery of partial object components may be reflected in brain wave dynamics, but specific feature information has yet to be identified, suggesting it may be best combined with methods such as fMRI. The detailed level of specificity accomplished through fMRI is limited by its lack of temporal resolution, whereas EEG is limited by spatial resolution.

Perceptual work conducted with fNIRS provides inconclusive evidence regarding hemodynamic responses as a reliable indicator of imagery-related activity. Although perceptual research employing fNIRS is still in its early stages, studies of object individuation and motor imagery suggest potential for future application of fNIRS to mental imagery events. Unfortunately, near-infrared is currently limited to the shallow cortical regions involved in visual processing, and the reliability of hemodynamic response for neural decoding remains to be validated. In order to accommodate the widely distributed and varied neural correlates of mental imagery formation, fNIRS should be combined with broader measurement instruments such as EEG or fMRI in order to aid effective indexing of visualized object features.

The methods and findings reviewed in this article are intended to support the feasibility and value of exploring perceptual diagnostic features through visual mental representations. Internal visual experiences, which occur in the absence of perceptual input, have the potential to be uniquely informative for understanding the manner in which visual information is transformed and translated to produce semantically meaningful object representations. By taking advantage of the natural information filtration processes imposed by the physical and cognitive limitations of human visual and neuronal systems, it is possible to access a great deal of semantic information via a condensed, concentrated source, in the form of distinguishing features. Mental imagery offers significant benefits over direct perceptual evaluations in that, during natural perceptual viewing, the potential amount of identifying object information is far greater than when an object is recalled through imagery alone, in part because irrelevant information has been discarded – or, at least, deemphasized – in mental representations. By studying object recognition solely as it occurs during perceptual processes, the researcher is forced to consider an inordinate amount of possibilities when determining the properties of a stimulus most essential to its identification. However, this procedure poorly reflects natural visual processes. By approaching visual perception from the level of its ultimate goal – the mental representation – and evaluating the relationship between the initial input and its final output, the investigator may reach a more complete and accurate understanding of the interaction between vision and cognition.

## Author Contribution

SR developed the concept, conducted the research, and wrote the text of this manuscript.

## Conflict of Interest Statement

The author declares that the research was conducted in the absence of any commercial or financial relationships that could be construed as a potential conflict of interest. The reviewer DL and handling Editor declared their shared affiliation, and the handling Editor states that the process nevertheless met the standards of a fair and objective review.
